# Removable denture is a risk indicator for peri-implantitis and facilitates expansion of specific periodontopathogens: a cross-sectional study

**DOI:** 10.1186/s12903-021-01529-9

**Published:** 2021-04-01

**Authors:** Jasmin Grischke, Szymon P. Szafrański, Uthayakumar Muthukumarasamy, Susanne Haeussler, Meike Stiesch

**Affiliations:** 1 Department of Prosthetic Dentistry and Biomedical Materials Science, Hannover Medical School, Carl-Neuberg-Str. 1, 30625 Hannover, Germany; 2 Lower Saxony Centre for Biomedical Engineering, Implant Research and Development (NIFE), Hannover, Germany; 3 Cluster of Excellence RESIST (EXC 2155), Hannover, Germany; 4 Institute for Molecular Bacteriology, TWINCORE GmbH, Centre for Clinical and Experimental Research, A Joint Venture of the Hannover Medical School and the Helmholtz Centre for Infection Research, Hannover, Germany

**Keywords:** Peri-implant mucositis, Peri-implantitis, Fixed dentures, Removable dentures, Prevalence, Risk indicator, Microbiome, Biofilm, Dysbiosis, *Prevotella intermedia*, *Fusobacterium nucleatum*, RNAseq

## Abstract

**Background:**

The prevalence of peri-implantitis ranges between 7 and 38.4% depending on risk indicators such as smoking, diabetes mellitus, lack of periodontal maintenance program, and history or presence of periodontitis. Currently, the possible effect of the type of superstructure on peri-implant health is unclear. This cross-sectional study aims to investigate the influence of the superstructure on the prevalence of peri-implant mucositis, peri-implantitis and peri-implant dysbiosis.

**Methods:**

During a 32-month recruitment period dental implants were assessed to diagnose healthy peri-implant tissues, mucositis or peri-implantitis. The study included 1097 implants in 196 patients. Out of all peri-implantitis cases 20 randomly chosen submucosal biofilms from implants with fixed denture (FD) originating from 13 patients and 11 biofilms from implants with removable dentures (RD) originating from 3 patients were studied for microbiome analysis. Composition of transcriptionally active biofilms was revealed by RNAseq. Metatranscriptomic profiles were created for thirty-one peri-implant biofilms suffering from peri-implantitis and microbiome changes associated with superstructure types were identified.

**Results:**

16.41% of the implants were diagnosed with peri-implantitis, 25.00% of implants with RD and 12.68% of implants with FD, respectively. Multivariate analysis showed a significant positive association on patient (*p* =  < 0.001) and implant level (*p* = 0.03) between the prevalence of peri-implantitis and RD. Eight bacterial species were associated either with FD or RD by linear discriminant analysis effect size method. However, significant intergroup confounders (e.g. smoking) were present.

**Conclusions:**

Within the limitations of the present work, RDs appear to be a risk indicator for peri-implantitis and seem to facilitate expansion of specific periodontopathogens. Potential ecological and pathological consequences of shift in microbiome from RDs towards higher activity of *Fusobacterium nucleatum* subspecies *animalis* and *Prevotella intermedia* require further investigation.

## Background

Dental implants are a valuable addition to the treatment options for partially and fully edentulous patients [1, 2]. However, in addition to technical complications such as ceramic chipping, screw loosening, fractures of the superstructure or the implant, dental implants may be colonized by diverse pathogenic microbial biofilms that strongly contribute to peri-implant complications. These polymicrobial infections can be distinguished clinically into two degrees of severity. The first stage of peri-implant inflammation, peri-implant mucositis, is comparable with gingivitis, an inflammatory disease of the soft tissues surrounding the natural tooth, and affecting only the peri-implant mucosa [3]. Without treatment the peri-implant inflammatory process may progress further into a second stage, peri-implantitis, and reach the peri-implant bone. Peri-implantitis is manifested by severe persistent infection, chronic destructive inflammation and may ultimately lead to implant loss [4].

Peri-implant mucositis (i) is an inflammation of the peri-implant soft tissues diagnosable by bleeding on probing (BoP) with or without increased probing depth, relative to baseline measurements, without bone loss exceeding the level of physiological crestal remodeling. Peri-implantitis (ii) is diagnosed by radiographic bone loss compared to earlier radiographs with clinical signs of inflammation such as BOP or suppuration with or without increased probing depth or mucosal recession [3]. The survival rate of dental implants ranges between 90 and 100% after 5 years post-implantation [5]. However, prevalence of peri-implantitis is 26.0% in patients with implant function time >5 years and incidence rises up to 43.9% within 5 years depending on risk factors [6]. The most effective measure to prevent peri-implantitis seems to be timely management of peri-implant mucositis [7]. Although the etiology of peri-implantitis has not yet been fully elucidated, the theory of infectious pathogenesis is widespread and universally accepted by the scientific community. This may also explain the increased risk of peri-implantitis in patients with periodontitis, inadequate oral hygiene and lack of participation in a regular recall [6]. Still, the histopathological, immunopathological and clinical factors (e.g. patient age, loading time, smoking status, gender, presence of keratinized mucosa) leading to the progression of peri-implant mucositis to peri-implantitis are far from being fully understood [8–10].

Peri-implant biofilm, a sessile consortium of diverse oral microorganisms, can play a decisive role in peri-implant physiology [11–13]. In healthy patients, there is a homeostasis between the host immunity and oral biofilms dominated by benign commensals. However, environmental, genetics, or physiological states can favor dysbiosis—microbial imbalance that cause or enhance pathological condition around dental implants. Understanding of ecological mechanism governing dysbiosis development and progression should therefore reveal molecular targets for future more personalized diagnostics and therapeutics [14–21]. Metatranscriptomics reveals transcriptionally active community in contrast to metagenomics that only uncovers genetics of microbiota [22]. It is more temporally dynamic, context-sensitive, and species-specific than the metagenome [23].

The superstructure is the part of the implant restoration penetrating the protective border of the peri-implant mucosa and is permanently exposed to colonization by the oral biofilms [24]. Different surface materials differently facilitate biofilm formation [25] and multi-species biofilms formed on the superstructure can serve as a reservoir for periodontopathogens that re-colonize the submucosal implant surface as significant transmission of microorganisms between teeth and implants, has been reported [26]. Additionally, different superstructures may provide different physicochemical conditions, e.g. removable dentures (RDs) when not worn exposes the implant to robust colonization and, when it is worn, dentures form a tightly closed thin chamber, likely enabling rapid development of anaerobiosis. Furthermore, reduced saliva flow can alter biofilm formation [27].

Rammelsberg et al. prospectively evaluated 1569 implants in 630 patients and concluded that the type of superstructure has an effect on implant prognosis, e.g. a tendency toward a greater incidence of complications for implants restored with RDs than for single crowns [28].

Accordingly, we examined implant patients to test the hypothesis, that RD is associated with higher prevalence of peri-implantitis and used metatranscriptomics to study the associated microbiota aiming to identify RD as a risk indicator for peri-implantitis.

## Methods

### Study design

This is a cross-sectional study aligned with the STROBE statement for cross-sectional studies. [29]

### Study population

The study participants were patients of the Department of Prosthetic Dentistry and Biomedical Materials Science of Hannover Medical School and were recruited between October 2016 and May 2019. Patients were approached during their regular treatment sessions and gave oral and written consent to participate in the study. The survey of the data and the inclusion of study participants took place in consideration of the ethics committee of Hannover Medical School (vote No. 3086-2016).

Only patients with at least one root-shaped titanium dental implant restored and in function for at least 12 months, were included in the study. Exclusion criteria were head or neck radiation during the last 6 months, antibiotics during the last 3 months, patients with a “heart passport”, immune-compromised patients, patients with uncontrolled diabetes, expecting or lactating mothers. The patient recruitment process and clinical assessment was performed by one examiner following a standardized protocol.

### Clinical examination

The collection of data was subject to special data protection measures, each patient was assigned a sequential number. All digital data were stored on the internal, secure and password-protected drives of Hannover Medical School. The examinations were carried out by an experienced practitioner to achieve standardization of the examination procedures, the probing pressure, the radiographic evaluation, the visual assessment and the subsequent diagnosis.

All implants were examined clinically and, when indicated, radiographically. Healthy implants without clinical signs of inflammation (BOP) were not examined radiographically. However, fistula, suppuration, bleeding on probing with an increase of PD or recession over time in relation to former examinations and pain were indications for radiographs. All patients that were diagnosed with peri-implant mucositis received radiographic examination to eventually diagnose peri-implantitis. The following parameters were collected:

i. age and gender, ii. bleeding on probing, iii. probing depth, iv. presence of periodontitis, v. history of periodontitis vi. smoking status, vii. residual teeth, viii. type of superstructure (fixed or removable), ix. full-mouth plaque index x. number of implants xi. implant age xii. type of implant. Additionally, submucosal plaque samples were collected. Clinical examination was performed with the help of a periodontal probe (CP15, Hu-Friedy Mfg. Co. LLC, Chicago, USA). Four aspects of each implant were assessed.

### Definition of outcomes

To evaluate the influence of the superstructure on peri-implant health, the exposure variable superstructure was categorized into (a) removable (RD) and (b) fixed dentures (FD). Based on the available information, one of the following diagnoses was made for each implant following the definition for peri-implant mucositis and peri-implantitis of group 4 of the World Workshop on Periodontal and Peri-implant disease [3]: i. healthy, ii. peri-implant mucositis, iii. peri-implantitis. Radiographs were obtained in case of clinical signs of inflammation to diagnose peri-implantitis. Peri-implantitis was diagnosed by radiographic marginal bone loss compared to earlier radiographs with clinical signs of inflammation such as BOP or suppuration with or without increased probing depth or mucosal recession. Radiographs were not standardized in terms of positioning and projection. The viewing platform was byzz (orangedental) with a minimum screen resolution of 1280 × 720. Specifically, we differentiated between peri-implant mucositis and peri-implantitis based on marginal bone loss measured in radiographs taken at different time points. An experienced examiner compared the marginal bone level of the current radiograph with former radiographs and captured dichotomously either there was radiographic bone loss (RBL) or not, however bone levels were not captured in mm.

Superstructures were defined as fixed dentures when they were cemented or screwed retained. Bar-supported, locator-supported, and telescope-prostheses were defined as removable. No distinction was made between overdentures and telescoping bridges.

Probing depth (PD) was measured using a periodontal probe (P 15, Hu-Friedy Mfg. Co., LLC., Chicago, USA). Measurements were taken at four implant sites (mesio-buccal, disto-buccal, mesio-lingual, disto-lingual). Peri-implant mucositis was evaluated utilizing the bleeding on probing index (BOP). If BOP occurred in one aspect, the whole implant was positive. Presence of periodontitis was defined at CPI (Community Periodontal Index) Code ≥ 3 [30]. Full-mouth plaque index (API) [31] was measured and oral hygiene was defined dichotomously either as sufficient or insufficient. An API ≥ 35% was considered as inadequate oral hygiene as described in [31]. Smoking status was either current smoker, former smoker or never smoker as described in [50].

### Plaque sampling and microbiome profiling

Submucosal plaque samples and samples of the peri-implant crevicular fluids were collected at six implant sites (mesial-bukkal, mesio-lingual, bukkal, disto-bukkal, disto-lingual, lingual) after drying the area. Samples were collected with the help of paper points (ISO 35/2.0, VDW GmbH, München, Germany) inserted in the peri-implant pocket for 30s and a titanium curette. Paper points were pooled for each implant and stored in RNA-protect at − 80 °C (Qiagen, Hilden, Germany) RNA was isolated based on previously described protocol [19]. Briefly, bacterial cells from biofilms collected by paper points and curette were lysed enzymatically in LM buffer (15 mg/ml of lysozyme and 500 U/ml of mutanolysin in 10 mM Tris–HCl with 1 mM EDTA and pH 8) at 25 °C and 350 rpm for 90 min. Next, samples were separated from paper points by centrifugation at maximal speed for 5 min through QIAshredder Mini Spin Columns (Qiagen). After adding RLT buffer (Qiagen) containing 1% β-mercaptoethanol, bacterial cells were mechanically disrupted by vortexing for 30 s in the presence of 50 mg sterile, acid-washed glass beads (diameter 106 μm; Sigma-Aldrich Chemie GmbH), which was repeated 10 times with at least 1 min intervals on ice. Total RNA was isolated using the RNeasy Mini Kit (Qiagen) according to the manufacturer’s instructions. DNA was digested with DNase I (Qiagen) in solution, followed by RNA purification according to the RNeasy cleanup procedure. Eukaryotic cytoplasmic and mitochondrial ribosomal RNA as well as bacterial ribosomal RNA was depleted with removal probes and magnetic beads using Ribo-Zero Kit Epidemiology (Illumina, San Diego, CA, USA) followed by ethanol precipitation. The quality and quantity of total RNA, enriched mRNA, and cDNA was assessed using the 2100 Bioanalyzer instrument and dedicated kits: RNA 6000 Pico and High Sensitivity DNA (Agilent Technologies). mRNA was converted to cDNA using ScriptSeq v2 RNA-Seq (Illumina). ScriptSeq Index PCR Primers (Illumina) were used to add barcodes. For sequencing, 15 ng of each library was used and 8–10 samples were multiplexed on a single lane. Cluster generation was performed with cBot (Illumina) using a TruSeq SR Cluster Kit v3–cBot-HS (Illumina). Samples were single-end sequenced for 50–68 cycles on an Illumina HiSeq 2500 sequencer using the TruSeq SBS Kit v3—HS (Illumina). Image analysis and base calling were performed using the Illumina pipeline v 1.8. Blank samples were included to control for potential contamination originating from paper points and reagents. Few samples with high ratio of human to microbial reads were re-sequenced to increase the sequencing depth. After clipping adapters and barcodes, rRNA was removed with SortmeRNA [32], remaining reads were blasted against human genome and human reads were removed. Non-ribosomal microbial RNA fragments were mapped genome wise against the HOMD reference sequences (the 461 annotated genomes) using the bowtie2 [33].

### Statistical analysis

The data obtained were transferred from the analogue diagnostic results to an Excel (Microsoft Corp., Redmond, USA) spreadsheet. The Excel spreadsheet was imported into SPSS, version 26 (IBM, Armonk, USA) and then a (1) descriptive analysis of the data was made, (2) the data were checked for normal distribution with the Kolmogorov–Smirnov (KS) test and histogram graphs (3) a multifactorial regression analysis was performed in Excel. A KS test and visual evaluation of histogram for 1097 samples confirmed a normal distribution of the data.

Contingency table analysis was applied to study the relationship between superstructure and diagnosis groups. Pearson Chi-Square statistics and adjusted residual values were calculated to identify associations.

Mean values of diagnoses (0 = healthy, 1 = peri-implant mucositis and 2 = peri-implantitis) were compared, using a two-sample t-test. Based on a univariate correlation analysis, possible cofactors were identified and considered for the multivariate adjusted model. In the next step, an adjusted sensitivity analysis was carried out, in which the parameters previously identified as cofactors were incorporated in the model. An adjusted regression analysis was performed.

PRIMER and PERMANOVA+, the suite of univariate, graphical and multivariate routines were used to analyze the microbiome data. Transcriptionally active microbiome profiles were screened for species associated with denture superstructures. We used blank samples and the correlation analysis to identify and remove the contaminating reads [20]. Briefly, for each peri-implant biofilm, after removing contaminating taxa, we created the relative abundance profiles for 309 oral species or subspecies including unnamed species-like human microbiome taxons (HMTs). Species were identified based on reads mapping to HOMD database [34]. Next, we removed data for rare and low abundant species that did not reach 1% in any of the samples. Both biofilm samples and 127 retained species were sorted using hierarchical cluster analysis performed on Bray–Curtis measures for each biofilm samples pairs and species pairs. Consequently, biofilms with similar profiles and co-occurring species can be easily localized in the shaded plot. The linear discriminant analysis (LDA) effect size (LEfSe) algorithm [35] was used to associate transcriptionally active species with denture superstructure types. To protect from Type I Error, LEfSe p values were adjusted with a Bonferroni correction that is a conservative approach given the number of 127 performed tests.

## Results

**Fig. 1:**
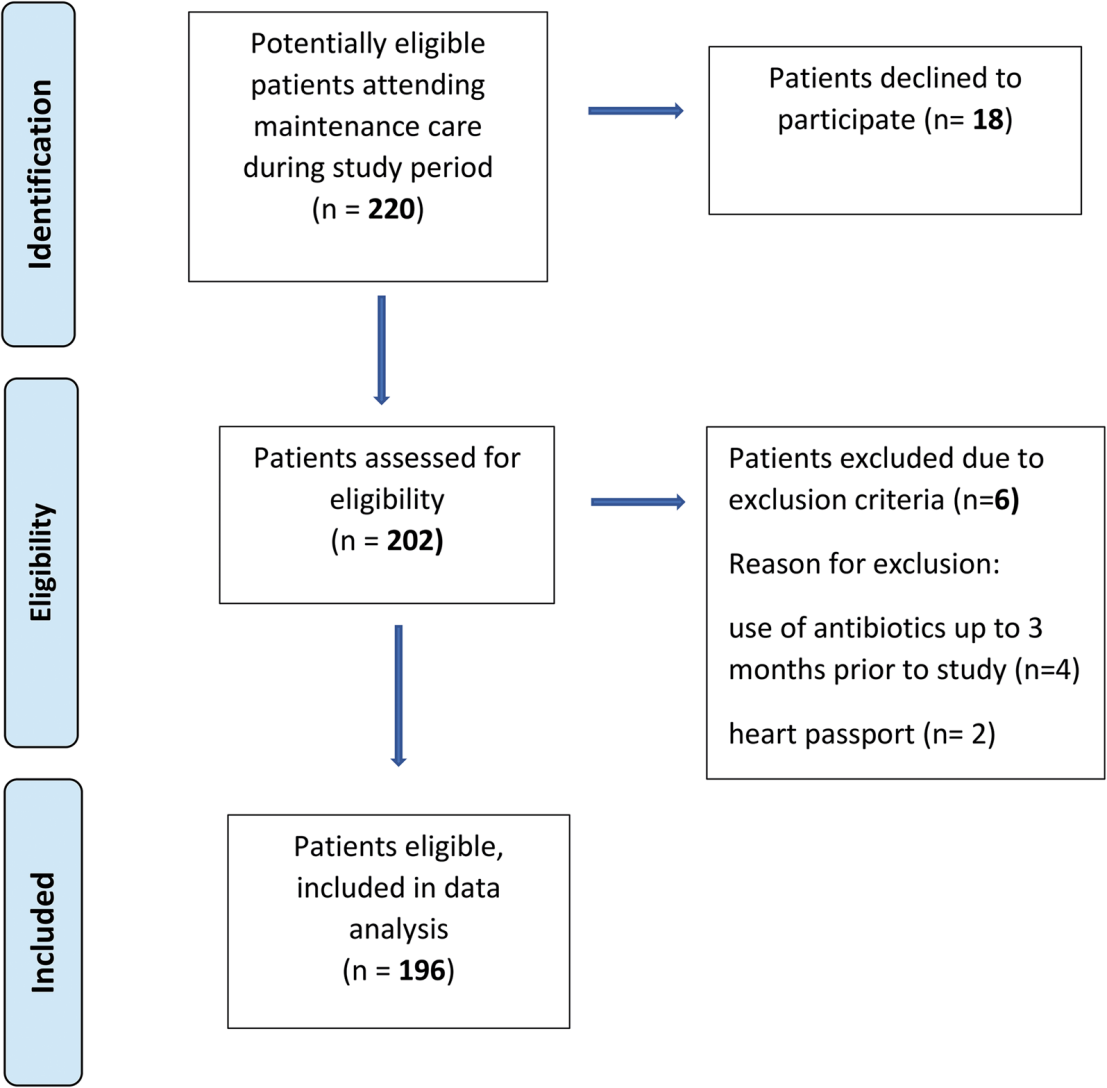
Flow-chart of patient recruitment depicting assessment of identification, eligibility and inclusion. Depicting numbers of and reason for exclusions

A total of 196 patients were examined. The recruitment process is depicted in Fig. 1. 104 female and 92 male participants with a mean age of 70.05 (± SD 9.47) years and with an age range between 26 and 92 years were recruited. The median age was 71 years. The implants were in situ for 9.86 ± 6.8 years with a range between 1–30 years. The mean PD was 3.96 ± 2.18 mm with a range between 1 and 15 mm.

Descriptive analysis revealed that 1097 implants were included in the cross-sectional study, of which 372 were diagnosed as healthy and 725 as diseased (Table 1). Peri-implant mucositis occurred in n = 545 implants and peri-implantitis in n = 180 implants. 69.74% (n = 765) of implants were restored with fixed and 30.26% (n = 332) with RD.

**Table 1:** Contingency table analysis. Counts, percentages, expected counts and adjusted residuals are given for prosthesis types and diagnosis groups

Type of prosthesis	Diagnosis	Health	Mucositis	Peri-implantitis	Total
FD	Count	283 (36.99%)	385 (50.33%)	97 (12.68%)	765
	Expected count	259	380	126	765
	Adjusted residual	3.3	0.6	− 5.1	
RD	Count	89 (26.81%)	160 (48.19%)	83 (25.00%)	332
	Expected count	113	165	54	332
	Adjusted residual	− 3.3	− 0.6	5.1	
Total	Count	372	545	180	1097

Peri-implant mucositis occurred in both types of dentures at a rate of about 50%. Peri-implantitis occurred roughly twice as often (25%) with RDs than with FDs (12.68%).

Contingency table analysis was performed using a Crosstabs function in SPSS on implant cases weighted by diagnosis frequencies and revealed that restoration type was associated with diagnosis (Pearson Chi-Square = 28.7, *df* = 2, *p* =  < 0.001, minimum expected count = 54). Low or high adjusted residuals values (< − 2 or > 2) for health and peri-implantitis but not for mucositis (RD: − 3.3, − 0.6 and 5.1, respectively) indicated that there were fewer health cases and more peri-implantitis cases than expected for RD group. [36]

In the group of fixed dentures, a mean value of 0.74 (mean diagnosis value with 0 = healthy, 1 = mucositis, 2 = peri-implantitis) was found. In the group of RD, a mean value of 0.96 with approximately the same variance was found. The t-statistic was − 4.48, indicating that peri-implant health was significantly better in the group of fixed dentures.

The two-sample *t* test, assuming equal variances (Table 2), showed a significant change in the dependent variable, peri-implant health, by the type of denture (p =  < 0.001). We have retrieved data on the type of implant of (brands: Astra, Straumann, Brånnemark and others) for 481 implants and found no significant influence on peri-implant health in our study sample (data not shown).

**Table 2:** Relationship between average diagnosis and type of prosthesis

	FD	RD
Average diagnosis	0.757	0.982
Variance	0.438	0.519
Observations	765	332
Pooled variance	0.463	
Hypothetical difference	0	
Degrees of freedom	1095	
t-statistics	− 5.034	
P(T < = t) one-way	** < 0.001**	
Critical t-value one-way t-test	1.646	
P(T < = t) two-way	** < 0.001**	
critical t-value two-way t-Test	1.962	

The two-sample *t* test, assuming equal variances showed a significant change in the dependent variable, peri-implant health, by the type of prosthesis (*p* =  < 0.001)

A two-way correlation analysis showed that there is a significant association (*p* = 0.01) between the type of superstructure (removable / fixed) and the diagnosis (healthy, peri-implant mucositis and peri-implantitis). Possible covariates that were not associated with our primary outcome in the univariate analysis were not included in the multivariate analysis. Consequently, the variables, which were found to be significant were used in the adjusted sensitivity analysis of the primary objective.

Using the implant as the statistical unit (Table 3), the multivariate model showed a significant association between the type of denture and the mean values of diagnosis (t-statistics 2.1668, 95% CI 0.0103–0.2081 *p* = 0.0304). Using the patient as the statistical unit (Table 4) and considering only one implant per patient (random implant), the adjusted analysis confirmed the findings on the implant level (t-statistics 3.7930, 95% CI 0.2341–0.7416, *p* < 0.001). The multiple linear regression on patient-level based on a binominal regression (peri-implantitis vs. no peri-implantitis) showed consistent results (Table 5). Additionally, implant age (*p* = 0.001) and oral hygiene (*p* < 0.001) were significantly associated with peri-implantitis.

**Table 3:** Results of the multivariate implant-level analysis

	Co-efficient	Standard error	t-statistics	p value	Lower 95%	Upper 95%
Superstructure	0.10	0.05	2.17	**0.03**	0.0103	0.21
Implant age	0.01	< 0.013	2.99	** < 0.01**	0.0032	0.02
Smoking	0.01	0.05	0.33	0.74	− 0.074	0.10
No implants	− 0.01	0.01	− 1.25	0.21	− 0.018	< 0.01
History of periodontitis	− 0.14	0.05	− 2.75	** < 0.01**	− 0.231	− 0.04
Sex	− 0.03	0.04	− 0.61	0.54	− 0.109	0.06
Presence of periodontitis	− 0.15	0.05	− 3.11	** < 0.01**	− 0.247	− 0.06
Residual teeth	0.16	0.07	2.22	**0.03**	0.0185	0.30
Oral hygiene	0.21	0.05	4.40	** < 0.01**	0.1189	0.31
Patient age	< 0.01	< 0.01	1.98	**0.05**	< 0.01	< 0.01

Table shows the results based on mean values for diagnosis (healthy = 0, peri-implant mucositis = 1, peri-implantitis = 2). Significant p-values are highlighted in bold

**Table 4:** Results of the multivariate patient-level analysis

	Co-efficient	Standard error	t-statistics	p value	Lower 95%	Upper 95%
Superstructure	0.50	0.13	3.79	** < 0.01**	0.23	0.74
Implant age	0.01	0.01	1.73	0.09	− 0.01	0.03
Patient age	− 0.01	0.01	− 0.10	0.92	− 0.01	0.01
Smoking	0.10	0.11	0.93	0.35	− 0.11	0.31
History of periodontitis	− 0.07	0.12	− 0.62	0.53	− 0.31	0.16
Sex	− 0.06	0.10	− 0.55	0.58	− 0.25	0.14
Presence periodontitis	0.03	0.12	0.22	0.82	− 0.21	0.26
Residual teeth	0.04	0.18	0.22	0.83	− 0.32	0.40
Oral hygiene	0.50	0.12	4.25	** < 0.01**	0.27	0.74
No°of implants	− 0.01	0.02	− 0.03	0.97	− 0.03	0.02

Table shows the results based on mean values for diagnosis (healthy = 0, peri-implant mucositis = 1, peri-implantitis = 2) for one random implant for each patient. Significant p-values are highlighted in bold

**Table 5:** Results of the multivariate patient-level analysis based on a binominal regression (peri-implantitis vs. no peri-implantitis)

	Co-efficient	Standard error	t-statistics	p value	Lower 95%	Upper 95%
Superstructure	0.38	0.14	2.68	** < 0.01**	0.10	0.65
Implant age	0.03	0.01	3.30	** < 0.01**	0.01	0.05
Patient age	− 0.01	0.01	− 0.12	0.90	− 0.01	0.01
Smoking	0.22	0.12	1.88	0.06	− 0.01	0.45
History of periodontitis	− 0.20	0.13	− 1.39	0.17	− 0.43	0.08
Sex	− 0.10	0.11	− 0.73	0.46	− 0.29	0.13
Presence of periodontitis	0.05	0.13	0.39	0.69	− 0.20	0.30
Residual teeth	0.04	0.20	0.19	0.85	− 0.35	0.43
Oral hygiene	0.56	0.13	4.38	** < 0.01**	0.31	0.82
No of implants	0.01	0.01	0.36	0.72	− 0.02	0.03

Significant *p* values are highlighted in bold

Secondary outcomes are significant associations between higher mean values of diagnosis and insufficient oral hygiene, higher implant age on patient-level as well as higher implant age, history and presence of periodontitis, edentulism, insufficient oral hygiene, and higher patient age on implant-level.

### Microbiome profiling

RNAseq-based biofilm profiles obtained with the small patient subgroup suggest that RDs favor expansion of specific periodontopathogens.

Clinical characteristics of samples selected for biofilm RNA-seq-based analysis is shown in Table 6. We studied 20 peri-implantitis biofilms from implants with fixed denture (FD) originating from 13 patients and 11 biofilms from implants with removable dentures (RD) originating from 3 patients. The location of implants was not balanced for both groups: e.g., front implants were more prevalent in RD. RD was also characterized by higher percentage of smokers, higher implant age, lower incidence of bleeding on probing, higher pocket depth, higher plaque index, higher incidence of bad oral hygiene, higher incidence of edentulism, lower patient age and higher incidence of “highly dysbiotic” community. In summary, cases from RD appeared as more severed diseased compared to cases from FD. We observed many factors apart from denture superstructure type that might contribute to pathology, e.g., location, age, hygiene, or smoking, however, we focused on the potential effect of dysbiotic microbiome.

**Table 6:** Characteristics of biofilm samples from implants with fixed and removable dentures

Types of prosthesis	Fixed dentures (FD)	Removable dentures (RD)	Test for significant difference
Number of implant	20	11	–
Numbers of individuals	13	3	–
Implant location: quadrants (1, 2, 3, 4)	9 (45%), 7 (35%), 2 (10%), 2 (10%)	3 (27%), 1 (9%), 5 (46%), 2 (18%)	n.s., Chi-Square Test
Implant location: maxillary	16/20 (80%)	4/11 (36%)	p = 0.015, Chi-Square Test
Implant location: left	11/20 (55%)	8/11 (72%)	n.s., Chi-Square Test
Type of replaced tooth (I, C, B, M)	0 (0%), 3 (15%), 6 (30%), 11 (55%)	4 (36%), 2 (18%), 4 (36%), 1 (9%)	-
Location of replaced toot: front	3/20 (15%)	6/11 (55%)	p = 0.020, Chi-Square Test
Implants from current smoker	0/20 (0%)	9/11 (82%)	p < 0.001, Fisher exact test
Implant age (µ ± 95%CI)	7.2 ± 1.7	12.4 ± 1.1	p < 0.001, Two-Tailed *T*-Test
Pocket depth (µ ± 95%CI)	6.3 ± 0.9	8.4 ± 1.6	p = 0.016, Two-Tailed *T*-Test
Gingival index (1, 2, 3)	0 (0%), 11 (58%), 8 (42%)	1 (9%), 6 (66%), 4 (36%)	-
Plaque index (0, 1, 2, 3)	1 (5%), 7 (37%), 6 (32%), 5 (26%)	0 (0%), 1 (9%), 1 (9%), 9 (82%)	-
Bad oral hygiene	5/16 (31%)	11/11 (100%)	p < 0.001, Fisher exact test
History of periodontal disease	20/20 (100%)	11/11 (100%)	n.s., Fisher exact test
Pus	10/20 (50%)	6/11 (55%)	n.s., Chi-Square Test
Pain	5/20 (24%)	1/11 (9%)	n.s., Fisher exact test
Periotron (µ ± 95%CI)	116 ± 22	161 ± 21	n.s., Two-Tailed *T*-Test
Residual teeth	13/13 (100%)	1/3 (33%)	p = 0.025, Fisher exact test
Patient sex: man	4/13 (30%)	1/3 (33%)	n.s., Fisher exact test
Patient age (µ ± 95%CI)	72 ± 3	63 ± 5	n.s., Two-Tailed *T*-Test

n.s.—the result is not significant at *p* < .05

**Fig. 2:**
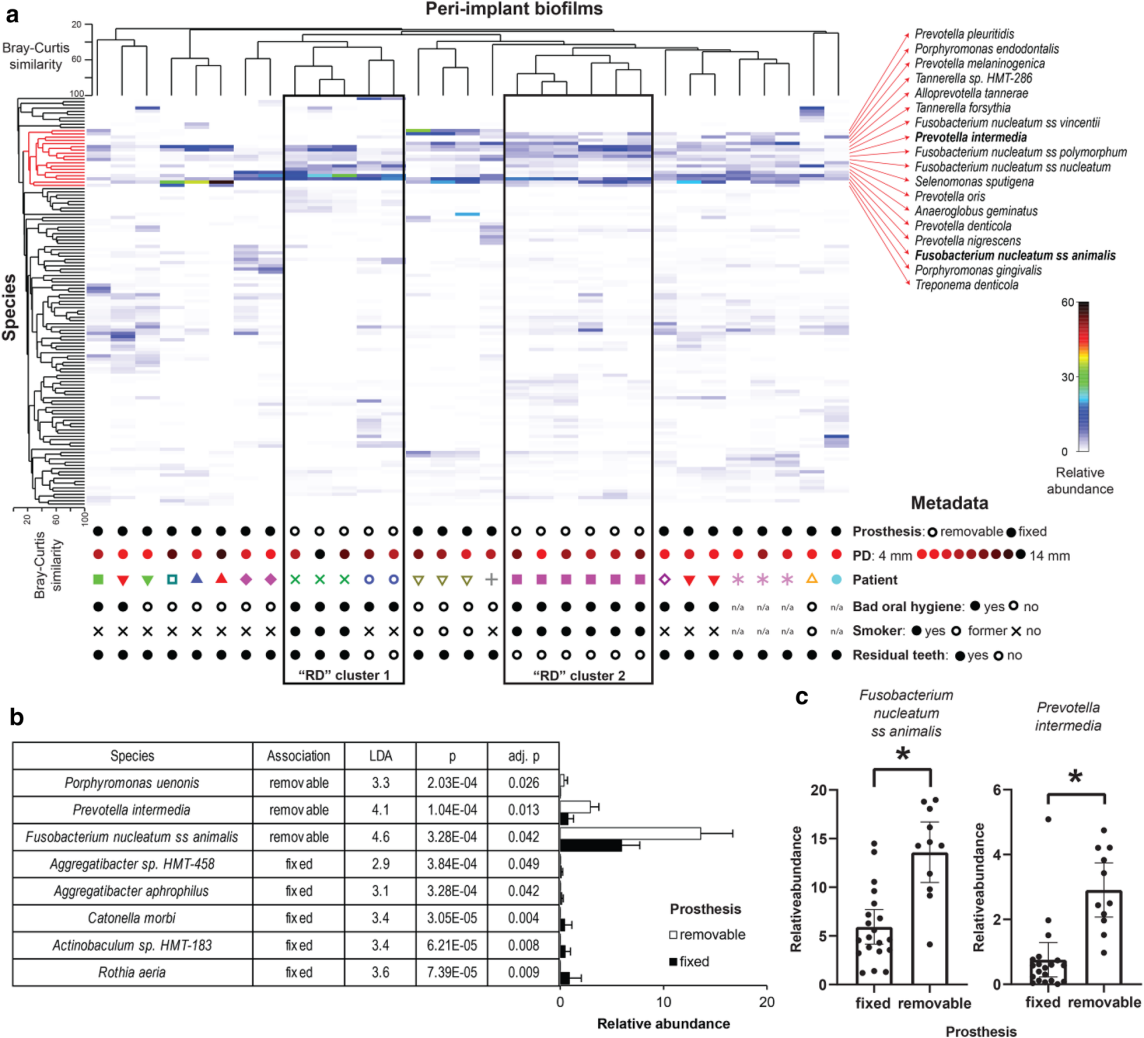
Biofilms on implants with removable (RD) or fixed prosthesis (FD) from patients with peri-implantitis. **a** Composition of transcriptionally active submucosal community. Cluster highlighted in red encompasses periodontopathogens. Metadata is given for each biofilm below a shade plot. **b** Characteristics of superstructure biomarkers. **c** Relative abundance of *Fusobacterium nucleatum* ss *animalis* (left) and *Prevotella intermedia* (right) in biofilms from implants with FDs or RDs assessed by RNAseq

Most of the peri-implantitis biofilms were dominated by well-known oral opportunistic pathogens (Fig. 2). Biofilms from the same patients clustered together. Cluster encompassing highly pathogenic species, indicated in red, contains two *Porphyromonas* sp., 4 *Fusobacterium nucleatum* subsp., 6 *Prevotella* or closely related *Alloprevotella* species. 2 *Tannerella* sp., *Treponema denticola, Selenomonas sputigena,* and *Anaeroglobus geminatus*. All these species have been associated with periodontal and/or peri-implant pathologies. We observed two separate clusters that grouped “RD” samples from three patients (Fig. 2a). Search for species that are differentially active on implants with FDs and RDs revealed 8 biomarkers (Fig. 2b). Among them two low abundant *Aggregatibacter* spp. and three other low abundant species were associated with “FD” while 3 taxons were characteristic for “RD” implants. Among these 3 taxons we found low abundant *Porphyrmonas uenonis* but also two highly abundant and ecologically important opportunistic pathogens, namely *Fusobacterium nucleatum* ss *animalis* and *Prevotella intermedia*. Distribution of relative abundance for two biomarkers with the biggest effect size is shown for two FDs and RDs in Fig. 2c, revealing a clear association pattern.

All sequencing data may be accessed at European Nucleotide Archive under study accession number PRJEB43417.

## Discussion

This cross-sectional study showed that the prevalence of peri-implantitis depends on the type of superstructure (fixed or removable dentures, abbreviated as FD and RD, respectively) in this cohort of university clinic outpatients. RD was identified as an independent risk indicator in the adjusted model. Scientific data on peri-implant health in direct comparison between RDs and FDs is scarce [6]. In line with the current literature we found RDs to be associated with higher prevalence of peri-implantitis compared to FDs [28]. We were able to identify RD as an independent risk indicator with a hazard ratio at 2.6 in the multivariate binominal regression analysis.

RNAseq profiling of submucosal biofilms in peri-implantitis revealed active microbial communities usually dominated by well-known oral opportunistic pathogens, e.g., *Fusobacterium nucleatum* subsp. animalis, *Porphyromonas gingivalis*, *Prevotella nigrescens*, *Tannerella forsythia* and *Fusobacterium nucleatum* subsp *vincentii* [41]*.* In some cases, biofilms were dominated by less studied species, often representing *Prevotella* genus, e.g., *P. pleuritidis*, *P. maculosa*, or *P. denticola* but the ecological role and pathogenic potential of this species is poorly explored [42]. We also observed in one of the patient the high transcriptional activity of *Cardiobacterium hominis*, a species that can cause infective endocarditis [43]. Thus, metatranscriptomic profiling can potentially identify implant carriers with higher risk of cardiac complications.

Hence, profiling reveals the active community members and can capture dynamic changes in biofilms. Two important periodontopathogens expended in microbiomes from implants with RD. *Fusobacterium nucleatum* and *Prevotella intermedia* are both associated with oral and extraoral pathologies, are equipped with numerous virulence factors, and have big impact on microbiome ecology [14, 19, 20, 44]. This would suggest that they may be directly or indirectly responsible for higher disease severity observed in patients with removable dentures. Alternatively, they are indicators of severe dysbiosis and tissue damage. Interestingly we observed that *Fusobacterium nucleatum* ss *animalis* and not the other subspecies were associated with removable dentures. This provokes a question about genetic determinants that drive superstructure specificity.

Interestingly, *Fusobacterium* species and subspecies were shown to differentially affect the composition and architecture of supra- and subgingival biofilms models, suggesting high functional diversity in *Fusobacterium* genus. [45] *Prevotella intermedia*, the other potential biomarker, commonly produces beta-lactamases and can resist higher concentrations of few antibiotics [46–48]. A variation in drug resistance patterns for peri-implant isolates representing abundant superstructure-associated species, suggests that molecular profiling of antibiotic resistance genes in peri-implantitis microbiota may further aid in the selection of antimicrobial therapy for peri-implantitis patients [47]. Distinguishing between closely related species (*Prevotella intermedia* sensu lato) or subspecies (the *Fusobacterium nucleatum* subspecies) can be challenging and studies addressing genomic diversity of these taxa could improve the genomic reference for microbiome studies. Organoid and animal models can be used to establish a casual effect of biofilm shift (towards specific periodontopathogens) on peri-implant tissue in context of superstructure type or implant material [49].

Meijer et al. (2014) summarized two prospective studies and found a 10-year incidence of peri-implant mucositis and peri-implantitis in edentulous patients with overdentures in 57% and 29.7%, respectively [37]. These numbers roughly correspond to the numbers found in the present work in the group of RDs (48% and 24%).

In a systematic review and meta-analysis, both current periodontitis [38] and history of periodontitis were identified as risk factor for peri-implantitis, which is in line with the findings in the present work [39]. We also found patients with residual teeth have significantly healthier implants. Interestingly, we found in our analysis that patients with no residual teeth are at elevated risk for peri-implant disease as described before in [6].

It may be assumed that the study cohort is representative for a university population. It would be advisable to perform a follow-on study that includes i. multiple centers, ii. private practice and focus on different populations in terms of e.g. geography, socioeconomic status and oral health behavior.

By including more than one implant of the same patient, pseudo replication may occur. The presented statistical evaluation considers this putative bias as we did patient-level analysis with one random implant for each patient and found consistent results. Furthermore, the clinical and microbiological status may strongly differ for implants in the same individual warranting implant-level analysis. A mixed-multi-level-model as described in [1] would be an alternative to account for patient-level clustering.

The prevalence of peri-implant mucositis (50%) and peri-implantitis (16%) both on implant- and patient-level observed in the present patient population correspond to the numbers published in recent reviews and the results on the patient level with only one implant per patient included in the analysis. Consequently, generalizability of the study results may be assumed, however subject to the limitations.

For microbiome analysis we analyzed only a small number of biofilm samples as a first pilot to investigate whether further research in this direction is promising. The metadata of the randomly selected patients shows that the implants in the RDs group are from (i) smokers and (ii) from only three individuals. This limits the conclusions that can be drawn from our findings as the effect may also be due to these cofactors. In future, it is necessary to validate the findings of the present microbial analysis in a more representative cohort in more individuals carrying RDs in a matched-case analysis.

Conclusively, microbiome analysis gave first insights into superstructure-specific effects on biofilms but bigger number of patients needs to be profiled to confirm our observations.

For future research the incidence of peri-implant disease in association with the type of superstructure for determining possible preventive aspects or the determination of success / or loss rates seems highly interesting. Consequently, the previously examined patients would have to be re-examined after a period of 5–10 years to elucidate the predictive value of peri-implant mucositis and the type of superstructure on the progression and initiation of peri-implantitis as the positive predictive value of peri-implant mucositis for the development of peri-implantitis has not yet been clarified until today [51].

Possible future approaches to investigate causality of the relationship between prevalence of peri-implantitis and RDs are quantitative and qualitative analyzes of the peri-implant bacterial biofilm and its changes over time.

## Conclusions

Peri-implant mucositis occurs at a rate of about 50%, regardless of the type of denture, while peri-implantitis is twice as likely (25.00%) for RDs than for FDs (12.68%). The hypothesis that the type of superstructure has an impact on peri-implant health must be accepted in this patient population as RD could be identified as an independent risk indicator for peri-implantitis in a model adjusted for co-factors. Potential role of shift in microbiome from RDs towards higher activity of *F. nucleatum* ss *animalis* and *Prevotella intermedia* and its therapeutic consequences require further investigation.

## Data Availability

The datasets analysed during the current study are available from the corresponding author on reasonable request.

## Abbreviations

RD: Removable dentures
FD: Fixed dentures
